# Floodplain ecohydrology: Climatic, anthropogenic, and local physical controls on partitioning of water sources to riparian trees

**DOI:** 10.1002/2014WR015581

**Published:** 2014-05-29

**Authors:** Michael Bliss Singer, Christopher I Sargeant, Hervé Piégay, Jérémie Riquier, Rob J S Wilson, Cristina M Evans

**Affiliations:** 1 Department of Earth and Environmental Sciences, University of St AndrewsSt Andrews, UK; 2 Earth Research Institute, University of California Santa BarbaraSanta Barbara, California, USA,,; 3 Université Lyon, UMR 5600—CNRS, Site ENS-Lyon, ISIG PlatformeLyon, France; 4 School of Mathematics and Statistics, University of St AndrewsSt Andrews, UK

**Keywords:** tree rings, oxygen isotopes (*δ*^18^O), Rhône, water partitioning, climate change, soil moisture

## Abstract

Seasonal and annual partitioning of water within river floodplains has important implications for ecohydrologic links between the water cycle and tree growth. Climatic and hydrologic shifts alter water distribution between floodplain storage reservoirs (e.g., vadose, phreatic), affecting water availability to tree roots. Water partitioning is also dependent on the physical conditions that control tree rooting depth (e.g., gravel layers that impede root growth), the sources of contributing water, the rate of water drainage, and water residence times within particular storage reservoirs. We employ instrumental climate records alongside oxygen isotopes within tree rings and regional source waters, as well as topographic data and soil depth measurements, to infer the water sources used over several decades by two co-occurring tree species within a riparian floodplain along the Rhône River in France. We find that water partitioning to riparian trees is influenced by annual (wet versus dry years) and seasonal (spring snowmelt versus spring rainfall) fluctuations in climate. This influence depends strongly on local (tree level) conditions including floodplain surface elevation and subsurface gravel layer elevation. The latter represents the upper limit of the phreatic zone and therefore controls access to shallow groundwater. The difference between them, the thickness of the vadose zone, controls total soil moisture retention capacity. These factors thus modulate the climatic influence on tree ring isotopes. Additionally, we identified growth signatures and tree ring isotope changes associated with recent restoration of minimum streamflows in the Rhône, which made new phreatic water sources available to some trees in otherwise dry years.

**Key Points:**

- Water shifts due to climatic fluctuations between floodplain storage reservoirs
- Anthropogenic changes to hydrology directly impact water available to trees
- Ecohydrologic approaches to integration of hydrology afford new possibilities

## 1. Introduction

An important challenge in ecohydrology is identifying the direct controls exerted by water or its absence on vegetation. It is particularly important to separate the influences of climatically driven water availability from local, site-based physical factors and anthropogenic impacts to the water cycle. This is possible using trees that record hydrologic signatures in their annual growth rings. Such knowledge would improve efforts to characterize past climate over large areas, to model catchment hydrology, and to predict growth responses to changing climate within individual trees, across forest stands, or even over broad regions of the globe. It could also be used to better understand the recent history of partitioning between water storage reservoirs (e.g., vadose versus phreatic zones). This would support drainage basin water management, as well as restoration of river flows and of forest resources. In this paper, we endeavor to disentangle the primary controls on water availability to trees rooted in floodplains of the Rhône River in France. We use historical time series of tree ring oxygen isotopes, ring width chronologies, instrumental climate data, high-resolution topography, and soil depth measurements to identify climatic, anthropogenic, and physical controls on spatial and temporal variability of water sources used by streamside trees and their influence on annual tree growth within this major river floodplain.

A basic premise here is that vegetation is dependent on the hydrologic cycle because water is fundamental to plant growth and functioning. Essentially, vegetation growth, especially for mature trees, responds to fluctuations in water availability and these response signals are detectable within vegetative tissues such as growth rings (see *Fritts* [1976] for an early review). Although ecological factors (e.g., competition between species, parasitism, infestation) influence tree growth both at the individual tree and stand level [*Fritts and Swetnam*, 1989], they can be straightforwardly assessed a priori and their influence on ring width series removed using flexible detrending approaches. Therefore, we may assume that for mature trees in temperate regions within a limited area and with no clear ecological disturbance, water availability is a first-order control on growth of trees, so long as variability of solar radiation, temperature, and relative humidity is low and stationary. An individual tree within such an area should therefore record annual information about its access to water with respect to climatic drivers (growing season soil moisture and/or water table elevation), as well as local conditions (depth of soil and elevation of roots above the water table). Moreover, if the hydrologic cycle has been affected by humans (e.g., extracting dry season irrigation water causing local water table decline or by impounding flow upstream), changes in water availability from particular hydrologic sources should also be evident within vegetation [*Amlin and Rood*, 2003; *Rains et al.*, 2004; *Snyder and Williams*, 2007].

The practice of reconstructing past precipitation and discharge regimes via tree rings has been widespread since the development and subsequent improvement of methods relating tree ring width to indices of wet and dry years [e.g., *Cook and Kairiukstis*, 1990; *Douglass*, 1920; *Fritts*, 1976]. These methods have been augmented in recent decades with the advent of isotopic analysis of oxygen and hydrogen in the source water used in the construction of tree rings (see *McCarroll and Loader* [2005] for a review). This has yielded insight into variability of past climate [*Burk and Stuiver*, 1981; *Edwards and Fritz*, 1986; *Epstein and Yapp*, 1976; *Gray and Thompson*, 1976; *Saurer et al.*, 2000] and the site-based conditions [e.g., *Dupouey et al.*, 1993; *Saurer et al.*, 1995,^1997^; *Snyder and Williams*, 2000; *Stromberg and Patten*, 1996; *Zencich et al.*, 2002] contributing to annual differences in isotopic signatures and tree growth. Several studies have used isotopes to identify water sources taken up by particular species [e.g., *Alstad et al.*, 1999; *Dawson and Ehleringer*, 1991; *Saurer et al.*, 2000], as well as to distinguish between water sources used by co-occurring species and to identify their temporal fluctuations [*Busch et al.*, 1992; *Marshall and Monserud*, 2006; *Singer et al.*, 2013; *Snyder and Williams*, 2000].

Oxygen isotopic ratios, *δ*^18^O, within tree ring cellulose reflect source waters used for tree growth, exchange with the atmosphere during transpiration, and the biochemical fractionation that occurs during photosynthesis [*McCarroll and Loader*, 2005]. In general, cellulose *δ*^18^O values are highly enriched by 27‰ compared with the source waters for all species [*Roden et al.*, 2000]. Although there is no fractionation of oxygen during uptake by roots [*Ehleringer and Dawson*, 1992], this high enrichment is associated with the formation of cellulose from sucrose in the incipient tree ring [*Sternberg and DeNiro*, 1983]. There is also variable fractionation in the form of evaporative enrichment within leaves (due to interactions between leaf water and atmospheric water vapor that depends on temperature, pressure, and water vapor). Ultimately, *δ*^18^O in tree ring cellulose contains a complex mixture of the signals of leaf-water enrichments and source water used by trees, so knowledge of the meteorologic conditions and the mixture of source water is required to constrain isotopic interpretations [*Barbour et al.*, 2004; *Roden and Ehleringer*, 1999; *Roden et al.*, 2000; *Waterhouse et al.*, 2002]. These factors may both vary annually as the climate fluctuates, in terms of precipitation, temperature, and relative humidity, and as the source water mixture available to trees shifts between different sources (e.g., precipitation, groundwater).

In order to improve the understanding of isotopic signatures in tree rings, some research has attempted to model the biochemical processes of atom exchange during photosynthesis in leaves and cellulose formation producing tree rings [e.g., *Barbour et al.*, 2004; *Flanagan et al.*, 1991; *Roden et al.*, 2000], based on isotopic fractionation theory applied to vegetation [*Craig and Gordon*, 1965; *De Niro and Epstein*, 1979]. One such model was employed using data from trees grown in controlled conditions and found a significant influence of relative humidity on tree ring cellulose for hydrogen and oxygen isotopes [*Roden and Ehleringer*, 1999,^2000^]. This work showed that leaf-water enrichment induces variability in relationships between isotopes within water sources and tree ring cellulose. Subsequently, *Barbour et al*. [2004] modeled the modulating influence of the Péclet effect on leaf water, the advection of unfractionated source water to the leaf opposed by the backward diffusion of evaporatively enriched water [*Farquhar and Lloyd*, 1993], and adapted the Roden model for *δ*^18^O in cellulose accordingly. Both of these modeling studies demonstrate that leaf-water enrichment is fundamental to cellulose isotopic composition, although there is general acknowledgement that it should be more pronounced in environments with low humidity and low precipitation [e.g., *Roden et al.*, 2005, Figure 2; *Farquhar et al.*, 2007]. Other studies have identified weaknesses in attempting to back calculate *δ*^18^O in cellulose considering only relative humidity, ring width, and temperature [*Anderson et al.*, 2002], suggesting that source water taken up by tree roots may be the more dominant signal contained within tree rings in environments where these climatic variables have low interannual variability.

When comparing co-occurring trees within the same study plot, the effects of relative humidity may be considered uniform for all trees at a site within a particular growing season, such that differences in tree ring isotopes can be defensibly considered to be a function of variable source waters used for tree growth. However, large interannual differences in relative humidity may mask source water switching between years [*Barbour*, 2007; *Roden et al.*, 2005]. We address this issue below (*Analysis of the Impact of Climate Variables on δ*^18^O) in order to isolate source water contributions to tree ring oxygen isotopes.

A major gap within most previous environmental tree ring isotopic research is the lack of characterization of hydrologic partitioning at the site scale, which may exert first-order controls on annual water availability to trees, the annually recorded isotopic signature, and corresponding tree growth [*Brooks et al.*, 2010; *Plamboeck et al.*, 1999; *Saurer et al.*, 1995]. Water content in the root zone, particularly within a riparian setting, is derived from various water sources depending on the dominant processes in the hydrologic cycle within a particular year (e.g., precipitation infiltration versus groundwater table rise), each of which may vary isotopically on a seasonal basis [*Brooks et al.*, 2010; *Roden et al.*, 2000]. Moreover, this hydrologic variability is not spatially uniform, depending on local physical conditions affecting water infiltration and retention, such as soil texture which impacts hydraulic conductivity [*Jarvis et al.*, 2013; *Plamboeck et al.*, 1999].

**Figure 1:**
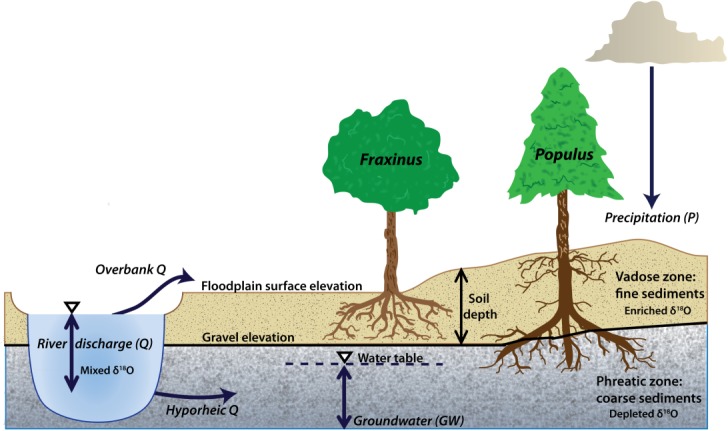
Schematic showing how two species with contrasting rooting depths may record different annual values of *δ*^18^O based on variability in hydrologic partitioning between floodplain storage reservoirs. In the Rhône case, *δ*^18^O in *Q* is generally very similar to that in *GW* because hyporheic flow dominates the alluvial aquifer. The controlling parameter space is likely to vary with relative elevation in the floodplain, as well as the depth to gravel.

Several physical variables germane to hydrologic partitioning, including local topography and soil depth, are poorly resolved for most forest locations, especially at the scale of individual trees. Yet they may have important influence over water availability and thus isotopic signatures of source waters to trees at various rooting depths. Local topography is a measure of how high a particular floodplain surface sits above the water table and therefore provides information on access to phreatic water. Soil depth (defined here as fine sediment thickness overlying gravel layers) determines how much water can be retained in the vadose zone and for how long after precipitation infiltrates. Within this domain, tree species have varying capabilities to grow roots that reach shallow groundwater aquifers. This has been demonstrated for *Populus* and *Fraxinus* trees in riparian zones [*Dufour and Piegay*, 2008; *Sánchez-Pérez et al*., 2008; *Singer et al.*, 2013]. Therefore, floodplain elevation of a particular tree above a particular river level may not be as important as gravel elevation below the surface for understanding potential access to groundwater in riparian floodplains, as well as the boundary between vadose and phreatic zones (Figure 1).

**Figure 2:**
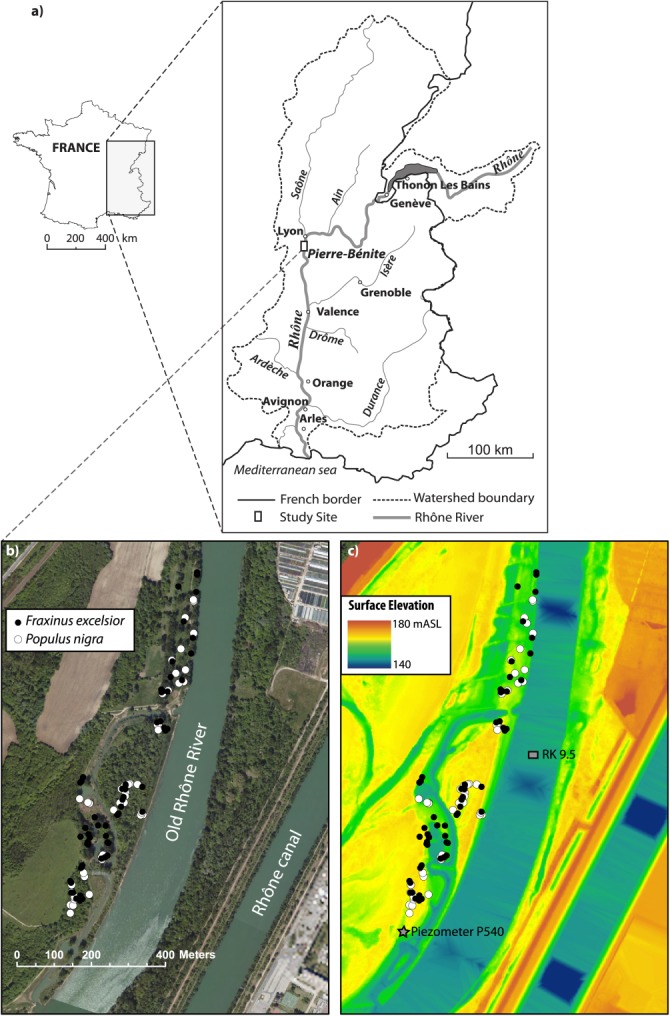
(a) Map of study area showing Pierre-Bénite within the great Rhône basin and France. Detailed maps of study site showing tree locations on an (b) aerial photograph and (c) LiDAR DEM backgrounds. The location of the piezometer used in Figure 6 is also shown, as is the River Kilometer used for the stage-discharge relationship in Figure 5. Source: BD Ortho©, BDT Rhône©, IGN.

Within the context of hydrologic variability and its nonuniform expression in the landscape, this study focuses on water availability and tree growth in the riparian corridor, which is typically characterized by an abundance of water available to streamside trees from various sources. Forested river floodplains are great environments in which to study ecohydrology because of strong connections between groundwater and streamflow [*Stromberg and Patten*, 1996], especially in terms of isotopic determination of water sources to trees [*Adams and Grierson*, 2001]. Although it has been shown for particular riparian sites that streamflow does not contribute significantly to water sources for trees [*Dawson and Ehleringer*, 1991], subsequent studies have shown that trees may use stream water, soil water, and/or groundwater [*Leffler and Evans*, 1999; *Singer et al.*, 2013; *Tang and Feng*, 2001; *Thorburn and Walker*, 1994; *Willms et al.*, 1998].

The water balance in floodplains consists of streamflow (*Q*), precipitation (*P*), groundwater (*GW*), which may be supported by hyporheic flow from the channel into the floodplain), evaporation from the soil, and transpiration from the root zone through leaves. Each of these hydrologic reservoirs has interannual variability, and if the end-member sources (e.g., *P* and *GW*) are isotopically distinct, there is potential for deconvolving their annual signatures in tree rings. However, most tree ring isotopic studies that address this hydrological partitioning have utilized only the current year's sap flow and xylem water measurements, leaving the historical record contained within tree rings unanalyzed [e.g., *Dawson and Ehleringer*, 1991; *Lambs and Muller*, 2002; *Sánchez-Pérez et al*., 2008; *Snyder and Williams*, 2000], although there are notable exceptions [e.g., *Anderson et al.*, 2002].

Using a multiparameter data set (tree ring and local water oxygen isotopes, tree ring width, instrumental climate data, high-resolution topography, and soil depth measurements), we investigate the variability in isotopic signatures within two co-occurring riparian tree species rooted at a range of floodplain elevations and with varying overbank fine sediment thickness at one site. We address several research questions: (1) how do trees in riparian zones access and use various water sources for growth through fluctuations in hydrology? (2) What role do relative floodplain elevation and soil depth play in controlling the partitioning of water in floodplain storage reservoirs? (3) How is tree water use and growth affected by climatic and/or anthropogenic changes in water source partitioning?

## 2. Study Area

This study is focused in the Rhône River valley of France at a site called Pierre-Bénite, situated ∼9 km downstream from Lyon and the confluence of the Rhône and the Saône River, a large tributary (Figure 2). The Rhône has a total length of 812 km with a catchment area of 98,500 km^2^ [*Olivier et al.*, 2009] and the study site is located ∼300 km upstream of the Mediterranean Sea. Like many European large rivers, the Rhône was regulated and impounded for navigation during the second half of the 19th century. This regulation was supported by groyne fields, which concentrate the flow into a single, straighter, deeper, and narrower main channel.

Many hydroelectric schemes were also built on bypass channels parallel to the main stem Rhône, which convey diverted flow from the main (natural) channel. Pierre-Bénite is located along one such 9.8 km bypassed section of what is now called the “Old” Rhône River (Figure 2), and which is now used to accommodate flood flows that exceed the maximum operating flow of the hydroelectric power plant (1365 m^3^ s^−1^), completed in 1966. The unimpaired mean annual discharge at this location is 1030 m^3^ s^−1^. In the dry season, only a residual discharge of 10 m^3^ s^−1^ between August and April and 20 m^3^ s^−1^ at all other time periods was flowing through the Old Rhône downstream from the hydroelectric dam, and this minimum discharge was mandated by the government until 2000 (see below).

The cumulative effects of groyne fields and hydroelectric infrastructure have impacted hydrological connectivity both between the main channel and a network of former secondary and backwater channels, as well as between the main Old Rhône channel and its floodplain, which has altered the river-floodplain ecosystem [*Klingeman et al.*, 1998; *Pautou et al.*, 1992; *Piégay et al*., 1997]. The relative average elevation of the floodplain above the restored minimum flow waterline for the 10th percentile flow is 3.8 m (±1.6 m), potentially stranding riparian ecosystems from a critical water source. As a response, a restoration project was initiated in Pierre-Bénite in the 1990s to improve the ecological condition of the local river corridor. In 1999, three floodplain channels were restored (dredged and/or reconnected to the “Old” Rhône) and July 2000, minimum flow in the “Old” Rhône was increased to 100 m^3^ s^−1^ [*Amoros et al.*, 2005] raising local groundwater level by an average of 0.5 m.

During the 19th century, the floodplain of the Rhône was extensively grazed and the forest was almost completely destroyed. The forest was re-established during the 20th century upon the fine-grained floodplain underlain by gravels, when grazing activity declined. In this riparian floodplain system, *Populus nigra* (black poplar, hereafter referred to as *Populus*) and *Fraxinus excelsior* (common ash, hereafter referred to as *Fraxinus*), the target species in this paper, are dominant tree species established at a wide range of floodplain elevations. These species have been interpreted elsewhere in the Rhône basin, to access distinct sources of water in many hydrologic years, due to their marked differences in rooting depth. Specifically, *Singer et al*. [2013] hypothesized for plots along the Ain River that *Populus* typically relies on phreatic water, which is generally assumed in prior work on *Populus* [e.g., *Rood et al.*, 2003]. However, during dry years, when it loses access to this water source, *Populus* must compete with *Fraxinus*, which subsists largely on vadose zone water. Since *Populus* has not invested in developing a strong dimorphic rooting structure [e.g., *Dawson and Pate*, 1996], its growth tends to suffer disproportionately during dry years when the regional water table drops. In other words, *Populus* use phreatic and vadose zone water [*Leffler and Evans*, 1999; *Singer et al.*, 2013], but *Fraxinus* trees are typically limited to water in the vadose zone because of their shallow root structure. The general balance of water contributing to the root zones of these species is illustrated in the schematic of Figure 1, which shows a shallow rooted tree (*Fraxinus*) accessing isotopically (evaporatively) enriched vadose zone water, and a deep rooted tree (*Populus*), with limited roots in the vadose zone, obtaining relatively depleted water from the phreatic zone. The figure also shows some potential changes to isotopic signatures in these floodplain water storage reservoirs based on annual partitioning.

## 3. Methods

### 3.1 Isotopic Characterization of Source Waters

**Figure 3:**
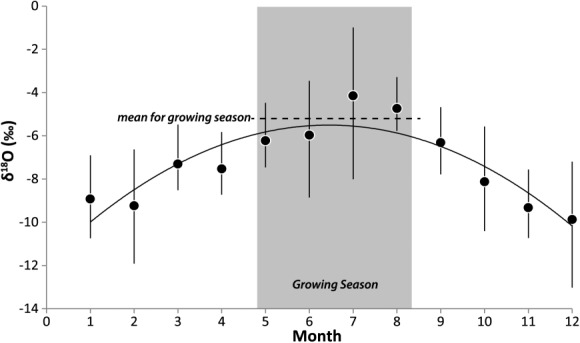
Monthly precipitation *δ*^18^O averaged between Avignon and Thonon Les Bains (Figure 2) from the Global Network for Isotopes in Precipitation (GNIP—http://www-naweb.iaea.org/napc/ih/IHS_resources_gnip.html), demonstrating a seasonal cycle of increasing *δ*^18^O during the growing season. Note: *δ*^18^O precipitation value of −5.3‰ is computed as an average of growing season monthly averages (shown as dashed line).

A seasonal cycle of *δ*^18^O in *P* has been observed in the Rhône basin, wherein oxygen isotopic signatures peak during the growing season (Figure 3). A regionally representative value of *δ*^18^O in precipitation for our study site was obtained by averaging the mean monthly values from the Global Network for Isotopes in Precipitation (GNIP) for the two nearest monitoring stations at Thonon Les Bains and Avignon (northeast and south of Pierre-Bénite, respectively, Figure 2), which we assume will best reflect the potentially different sources of growing season (May-August) precipitation in the basin (Atlantic versus Mediterranean). The averaged values of *δ*^18^O in *P* thus obtained yield an overall regional growing season *δ*^18^O of −5.3‰. These *P* measurements can be compared with our measurements of *δ*^18^O in Rhône *Q* (−10.4‰ in September 2012 at Pierre-Bénite and −10.6‰ for July 2013 at Donzère, north of Orange, Figure 2). Water samples were collected in sealed tubes with no head space and kept cool until laboratory analysis (see below). These values are largely consistent with other measurements of river water along the Rhône and Saône Rivers upstream of Pierre-Bénite before and after the 2002 growing season, which were made in a previous study [*de Benedittis and Bertrand-Krajewski*, 2005]. We computed a weighted average of these local 2002 measurements based on mean annual *Q* of each river (600 m^3^ s^−1^ versus 470 m^3^ s^−1^ for the Rhône and Saône, respectively), which was −9.5‰. *de Benedittis and Bertrand-Krajewski* [2005] also measured shallow *GW δ*^18^O along these two rivers yielding a weighted average of −9.3‰, which suggests phreatic water is largely composed of river water in the growing season.

In general, groundwater aquifers are often depleted isotopically, compared with river water [e.g., *Alstad et al.*, 1999; *Sklash et al.*, 1976]. Although groundwater isotope signatures are typically constant through time, within any particular basin they are dependent on a range of factors that transform the isotopic values of the prevailing precipitation [*Gat and Tzur*, 1968]. In a riparian zone, streamside shallow groundwater reservoirs may be affected by infiltration from hyporheic streamflow at rates much higher than from infiltrating precipitation, resulting in a relatively depleted water source in the phreatic zone. Generally, Rhône River water (often depleted isotopically because it is derived from snowmelt) fills up its alluvial aquifer via hyporheic flow as discharge increases associated with snowmelt at the beginning of the growing season and subsequently, this water drains back into the Rhône at the end of the spring snowmelt season. Therefore, isotopic values in the shallow groundwater are very similar to those of Rhône River water [*de Benedittis and Bertrand-Krajewski*, 2005; *Schürch and Vuataz*, 2000], yet they may deviate in particularly low *Q* years when hyporheic discharge into the alluvial aquifer is minimal. In such years, the primary water source available to riparian trees would either be relatively depleted groundwater remaining in the phreatic zone from previous years (for trees that can reach it) or relatively enriched vadose zone water from precipitation. In summary, average growing season *δ*^18^O in *P* is at least 4‰ higher than *Q* and shallow *GW*, providing excellent potential for discerning between water sources to riparian trees along the Rhône, assuming that the factors that modulate leaf water evaporative enrichment across the site are an order of magnitude lower than the source water differences.

### 3.2. Tree Ring Growth and Isotopic Characterization

We obtained 5 mm diameter tree cores using a Swedish increment borer (two per tree) from *Populus* (*Populus*) and *Fraxinus* (*Fraxinus*) within a near-channel floodplain containing a range of elevations and sediment thicknesses. Visually healthy, mature (mean age >30 years and mean diameter breast high (DBH) >20 cm) trees were selected in order to remove any potential ecological impacts to tree growth or water sources that would affect specific age cohorts. It may be reasonable to assume that our relatively young trees would have annual tree ring isotopic ratios that reflect the different water sources in the floodplain associated with progressive growth of roots as they approach maturity (e.g., a juvenile effect), rather than climatic variations in water sources. However, any juvenile effect on root growth for these riparian species within our study site is undoubtedly short lived because such trees grow very rapidly in the presence of abundant water and nutrients and favorable soil texture that are characteristic of perennial temperate river floodplains [*Dufour and Piegay*, 2008; *Lambs et al.*, 2006; *Rood et al.*, 2003; *Sánchez-Pérez et al*., 2008; *Stella et al.*, 2012]. For example, mean vertical root growth = 2.5 cm/d for Populus seedlings [*Mahoney and Rood*, 1998] in riparian environments, suggesting that roots would penetrate ∼2 m deep in only three growing seasons and would therefore be fully developed over a decade. For our sampled trees in each cohort, the average age at the first year used for isotopic analysis was 16 years for *Fraxinus* and 11 years for *Populus* and the first individual year analyzed (for rainfall versus snowmelt year comparisons) occurred 5 years later, rendering these trees 21 and 16 years old, respectively. By this point in their growth history, both of our sampled riparian species would be expected to have fully developed roots. Therefore, our analyses of oxygen isotopes on highlighted years of inquiry should not be biased by any juvenile effect that might impact their access to particular water sources, which is consistent with prior work at the tree line demonstrating a lack of the juvenile effect on carbon isotopes [*Daux et al.*, 2011].

For this study, we utilized 59 *Fraxinus* and 51 *Populus* trees that were all located <200 m from the Old Rhône (Figures 2b and 2c). Cores were visually cross dated and ring widths were measured under a microscope using the Measure J2X Tree-Ring Measuring Program. Cross dating was verified using cross-correlation analysis in the quality control program, COFECHA [*Grissino-Mayer*, 2002]. A 30 year spline was used to remove the age-related trend in each ring width series allowing only the retention of decadal and higher-resolution variability in the time series [*Cook and Peters*, 1981]. However, this frequency bias does not impact the aims of this study as we are interested only in the tree-growth response to water availability at higher frequencies.

We extracted *α*-cellulose from the raw whole wood of individual dated tree rings (combined earlywood and latewood) by a modified version of the Brendel method [*Brendel et al.*, 2000], which speeds up extraction and allows for small cellulose samples, while still yielding good results compared to other methods [*Evans and Schrag*, 2004]. We present isotopic data for 50 trees (one core was analyzed for any particular tree, but not all trees were used in cohort comparisons—see below), which we subdivide by species, as well as by floodplain elevation, gravel elevation, and soil depth. These cellulose samples were subsequently analyzed at the University of St Andrews to obtain oxygen isotopes in a Finnigan Delta plus XP gas source isotopic ratio mass spectrometer (IRMS), coupled by continuous flow to a Thermo Finnigan High Temperature Conversion/Elemental Analyzer (TC/EA) peripheral operated at 1350°C for cellulose pyrolysis. Water samples were analyzed using a Thermo Finnigan Gasbench II peripheral to the IRMS following CO_2_ equilibration. The precision (uncertainty) for 30 replicate *δ*^18^O measurements in *α*-cellulose (certified reference material IAEA 601: +23.3‰ Vienna Standard Mean Ocean Water or VSMOW) over 10 IRMS runs was 0.16‰. The precision value for our internal lab reference material (cellulose from a nearby paper mill: +31.05‰ VSMOW) was 0.10‰ for 56 measurements over 10 runs. Oxygen isotopic ratios (presented in units of ‰) were calculated as: 
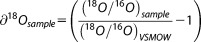
where (^18^*O*/^16^*O*)_*VSMOW*_ = 2.005 × 10^−3^ and isotopic data were corrected against IAEA standards.

### 3.3. Analysis of the Impact of Climate Variables on δ^18^O

In order to quantify how atmospheric conditions might affect our isotopic values in tree ring cellulose, we investigated the role of interannual differences in relative humidity (RH), temperature (T), and barometric pressure by first extracting daily values of these climate variables (individual values downloaded from the Global Summary of the Day-GSOD, http://www.climate.gov/global-summary-day-gsod at the station of Lyon near Bron, 074800-Latitude: 45.717°N/Longitude: 004.933°E) for the 3 month growing season, 15 May–15 August. We computed mean and standard deviation (SD) values of RH, T (supporting information Figure S1), and pressure for each year's growing season. For our site over the period 1990–2010, mean growing season values (±1 SD) were as follows: RH = 58.3% ± 4.2%; T = 20.1°C ± 1.0°C; air pressure = 101.6 kPa ± 1.0 kPa (note: pressure data were unavailable for the years 1992–1998). We investigated the impact of interannual differences in atmospheric variables on *δ*^18^O composition in tree ring cellulose by first plotting mean isotopic compositions in tree ring cellulose for both species against RH and there was no visible or statistical trend (supporting information Figure S2). Next we employed the mechanistic Barbour model [*Barbour et al.*, 2004], which builds on that of *Roden et al*. [2000], accounting for exchange between leaf water and meteoric water, while also incorporating the Péclet effect [*Farquhar and Lloyd*, 1993]. We hindcasted annual tree ring cellulose values based solely on our computed average growing season variations in RH and T, while keeping source xylem water input constant separately for characteristic *δ*^18^O in precipitation (−5.3‰) and in groundwater (−9.5‰). Atmospheric water vapor *δ*^18^O was computed for varying air temperatures (T) based on equilibrium fractionation in *δ*^18^O between average precipitation (−5.3‰) and water vapor [*Majoube*, 1971]. Our goal in this sensitivity analysis was not to accurately predict cellulose isotopic signature, rather to assess the role of climatic conditions (i.e., T and RH) on it. All other model parameters were kept constant. We obtained characteristic regional conductance/transpiration rates for *Fraxinus* (0.2 mol m^−2^ s^−1^/3.9 mmol m^−2^ s^−1^ [*Lemoine et al.*, 2001]) and *Populus* (0.278 mol m^−2^ s^−1^/4.73 mmol m^−2^ s^−1^ [*Lambs et al.*, 2006]). Other constants within the Barbour model included: effective path length computed based on a regression against transpiration rate for multiple tree species provided by *Song et al*. [2013]; proportion of exchangeable oxygen in cellulose = 0.42 [*Roden et al.*, 2000]; and proportion of xylem water in meristem = 1. All computations and results from this analysis are contained within the supporting information.

For the characteristic precipitation water source (*δ*^18^O = −5.3‰) and varying RH over one SD (54.1–58.3%) as a sensitivity analysis, *δ*^18^O in tree ring cellulose differed by 0.90‰ and 0.92‰ for *Populus* and *Fraxinus*, respectively, while for the groundwater source (*δ*^18^O = −9.5‰) RH caused cellulose *δ*^18^O to vary by 0.80‰ and 0.82‰, respectively. The difference in *δ*^18^O between the two species was 0.15‰ for mean RH under both *P* and *GW* isotopic sources. These values of tree ring cellulose *δ*^18^O difference, associated with variability in RH, remain constant even for warmer and cooler T (varying T over 1 SD in either direction). In contrast, the mean value of *δ*^18^O difference in tree ring cellulose associated with changing the assumed end-member water source isotopic composition (from *P* to *GW*) is −3.02‰ for both *Fraxinus* and *Populus*, several fold higher than the variability in modeled tree ring *δ*^18^O induced by the small measured variability in RH. Thus, since climate variables can be accounted for in the model for each growing season (via the GSOD data set), we can defensibly investigate source water variations associated with hydrologic partitioning in the floodplain.

### 3.4. Ancillary Variables and Hydrologic Characterization

To assess floodplain thickness of the fine sediment layer overlying the gravel layer (hereafter referred to as soil depth), we used a cone penetrometer at the base of each tree (i.e., depth to gravel based on first refusal). We employed high-resolution (2 m horizontal, average vertical precision of 20 cm) LiDAR data from 2010 (Figure 2c) to extract floodplain surface elevation and to compute the elevation of the gravel layer, by subtracting the soil depth from the surface elevation. We then analyzed tree ring growth chronologies, *δ*^18^O in tree rings, alongside decadal hydrologic records of *Q*, *P*, and water table elevation from a local piezometer (Figure 2c).

To isolate the partitioning of water in the floodplain, we focused the study on the 1990–2010 period, within which we have a roughly equal number of years to analyze on either side of the flow restoration and we can well constrain ancillary climatic variables. In order to assess water partitioning between floodplain storage reservoirs available to trees rooted at different positions (Figure 1), we segregated our data by species and into growing position end-members (cohorts) such that we could compare growth and isotopic values associated with, for example, differences between trees rooted at high (>158 mASL) versus low (<157 mASL) relative floodplain elevations. We maintain this high versus low elevation terminology throughout in order to indicate relative floodplain elevations above an arbitrary river level. Furthermore, we assume a dependence on underlying substrates through which water must pass into the root zone. Therefore, we also subdivided our tree ring data into end-member soil depths (<1 m versus >2 m), which allowed us to compute the gravel elevation below each tree (<155 mASL versus >156 mASL), indicative of the boundary between phreatic and vadose zones (Figure 1).

We use the term cohorts of trees here to indicate groups of trees that would be expected to behave similarly from the perspective of hydrologic partitioning in the floodplain. For example, *Populus* trees at relatively low floodplain elevations can presumably access deeper sources of water than those rooted at high elevations, so they represent their own cohort. For all intraspecific and interspecific comparisons between cohorts, the Kolmogorov-Smirnov (K-S) test, a two-tailed nonparametric test, was used to assess whether two groups of trees are drawn from the same distribution. Results for K-S tests are presented when they were significant at *p* < 0.05. This test does not quantify whether one cohort has larger average values (of growth or *δ*^18^O) than the other; it merely tests the similarity of their sample distributions. Therefore, in cases where K-S tests between cohorts were significant, we further tested for significant differences in the medians of the distributions via the Wilcoxon rank sum test and indicate the direction of these differences in summary tables. We also computed significant differences in grand means between cohorts for individual years based on no overlap of confidence intervals two standard errors around the mean, which are presented in relevant figures (Figures 10) as stars near the *x* axis. Finally, we used correlation analysis to determine whether different cohorts behave together in time series (sequences of years) and these results are presented in the text.

**Figure 4:**
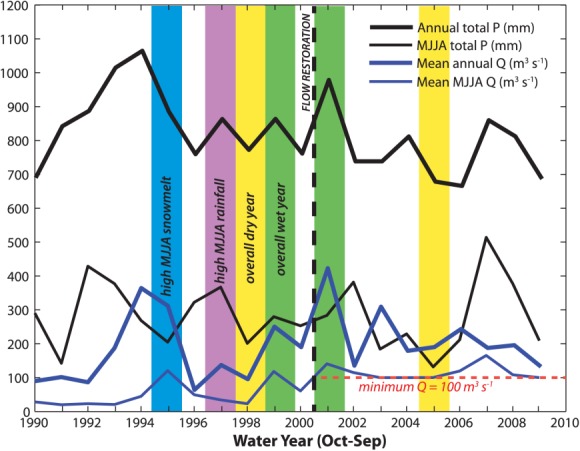
Last 20 water years (September-October) of total precipitation and mean annual discharge data, including growing season (May-August) values. Vertical bars highlight distinct years in the record when we might expect differences in water availability and thus oxygen isotopes in tree rings.

**Figure 5:**
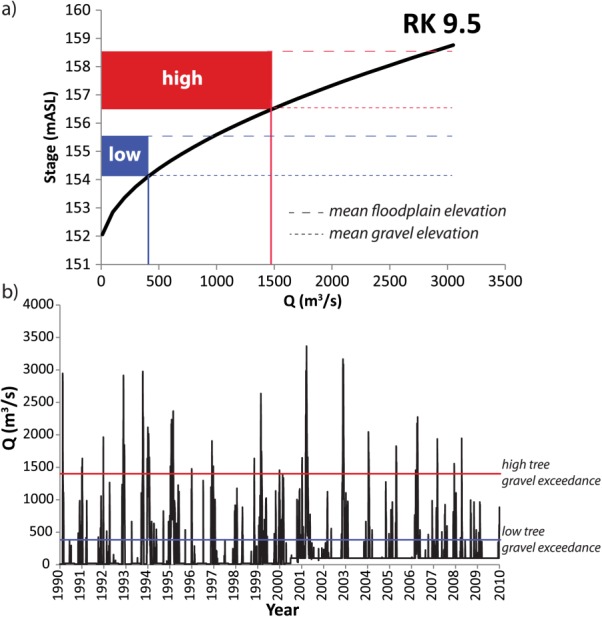
(a) Stage-discharge relationship for River Kilometer (RK) 9.5 along the “Old Rhône” river channel (Figure 2). “High” and “low” trees elevation range is indicated in red and blue, respectively. The dashed line indicates the average floodplain surface elevation and the dotted line marks the average gravel layer elevation, as determined by penetrometer. (b) Daily discharge data for the study period (provided by the Compagnie Nationale du Rhône). Discharges required to exceed the gravel elevation for high and low trees are indicated (based on stage-discharge curve in Figure 5a).

**Figure 6:**
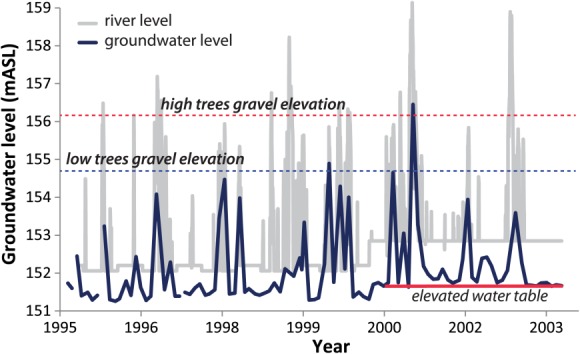
Piezometer (P540, Figure 3) and river stage data (provided by the Compagnie Nationale du Rhône) illustrating offset between river level and floodplain water table elevation. Average gravel elevation, measured via penetrometer, is also depicted for high versus low trees. The elevated water table since 2000 is due to flow restoration.

We processed the *P* and *Q* data (provided by the Compagnie Nationale du Rhône) to obtain characteristic series that correspond to the growing season for these species (May-August, “MJJA” in Figure 4) and we identified particular hydrologic years for cross comparison of isotopes and growth. Specifically, we selected characteristic wet (total *Q* ≥ 275 m^3^ s^−1^ and total *P* ≥ 850 mm yr^−1^) and dry (*Q* ≤ 200 m^3^ s^−1^ and *P* ≤ 800 mm yr^−1^) years before and after the flow restoration. In addition, we sought to isolate the differential effects of a year with high snowmelt runoff but low spring rainfall (MJJA *P* ≤ 200 mm yr^−1^ and MJJA *Q* > 100 m^3^ s^−1^) versus one with high spring precipitation but low spring discharge (MJJA *P* > 350 and MJJA *Q* ≤ 100 m^3^ s^−1^; Figure 4). We acknowledge that the selected years for these comparisons may not be the most representative of such climatic variation throughout the entire climatic record, but they are indicative of an iteration of identifiable climatic differences that we expect to impact hydrologic partitioning. The null hypothesis is that such climatic differences have not impacted isotopes or growth. We also obtained daily *Q* data and a stage-discharge rating curve (Figure 5) for the KM 9.5 location (Figure 2c), as well as piezometer data to assess the water table elevation of shallow *GW* within the floodplain with respect to flow in the channel (Figure 6).

**Figure 7:**
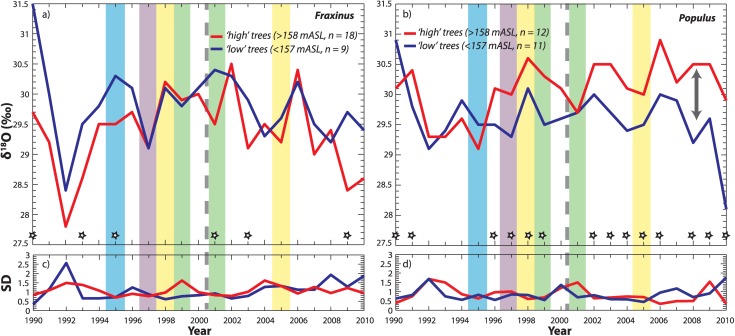
Mean oxygen isotope ratios, *δ*^18^O, for high versus low trees for two co-occurring species: (a) *Fraxinus excelsior* and (b) *Populus nigra*. For Figures 10, the following apply. The year 2000 flow restoration is indicated, as well as individual years under closer scrutiny in this study, where the color coding corresponds to that in Figure 4. Stars near the *x* axis indicate significantly different values between the grand means of cohorts for individual years (based on two standard errors around the mean). *N* values listed in the legend are based on the number of trees used to compute cohort means.

## 4. Results

**Figure 8:**
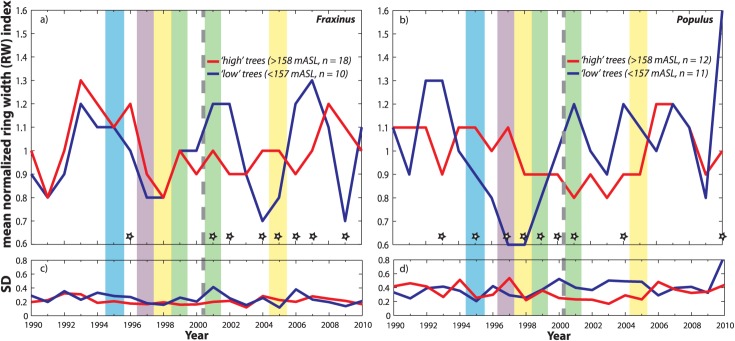
Mean detrended, dimensionless ring width index (based on 30 year spline fit) for high versus low trees for two co-occurring species: (a) *Fraxinus excelsior* and (b) *Populus nigra*.

**Figure 9:**
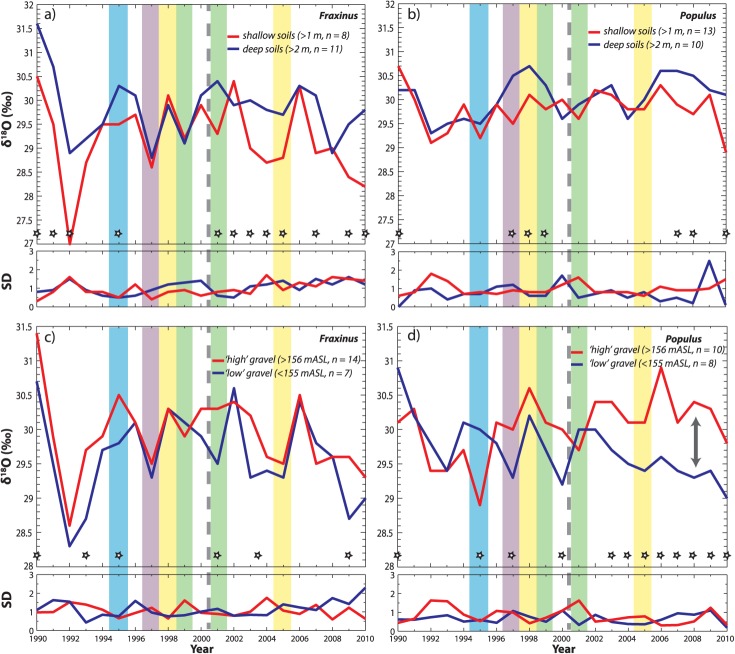
Mean oxygen isotope ratios, *δ*^18^O, for (a) *Fraxinus* and (b) *Populus* rooted deep versus shallow soils (measured by penetrometer to first refusal). (c and d) Mean *δ*^18^O for “high” versus “low” gravel elevations determined by subtracting penetration depths from floodplain surface elevation from determined from LiDAR.

**Figure 10:**
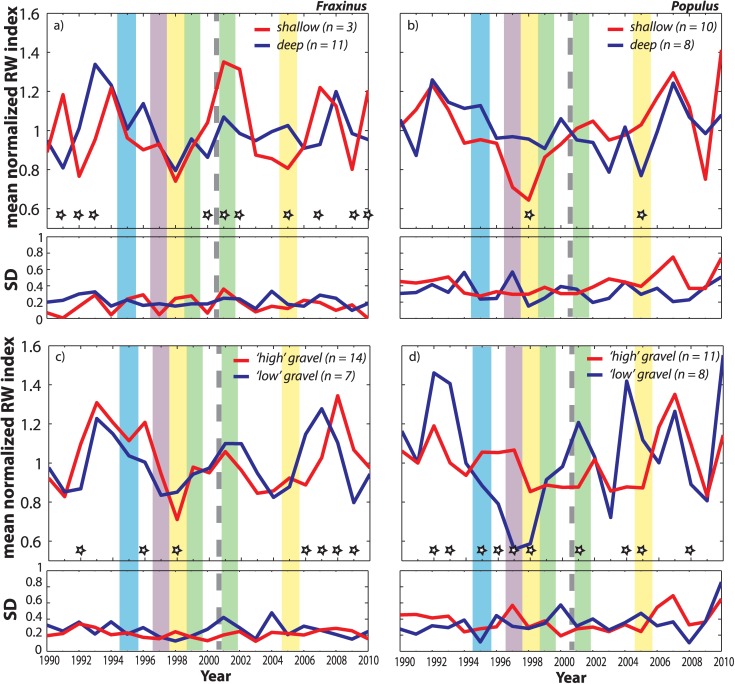
Mean detrended, dimensionless ring width index (based on 30 year spline fit) for (a) *Fraxinus* and (b) *Populus* rooted in deep versus shallow soils (measured by penetrometer to first refusal). (c and d) Mean growth for each species rooted at “high” versus “low” gravel elevations determined by subtracting penetration depths from floodplain surface elevation from determined from LiDAR.

**Figure 11:**
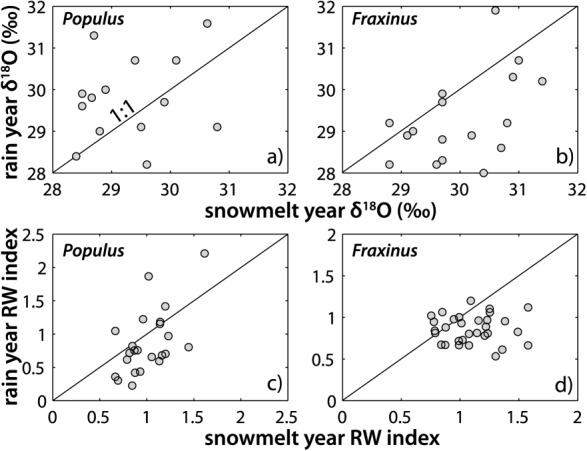
Individual oxygen isotope ratios and growth for each species in high spring snowmelt versus high spring rain years (see Figure 4). (a) *Populus δ*^18^O; (b) *Fraxinus δ*^18^O; (c) *Populus* growth; and (d) *Fraxinus* growth. Supporting statistics are provided in Table 1.

**Figure 12:**
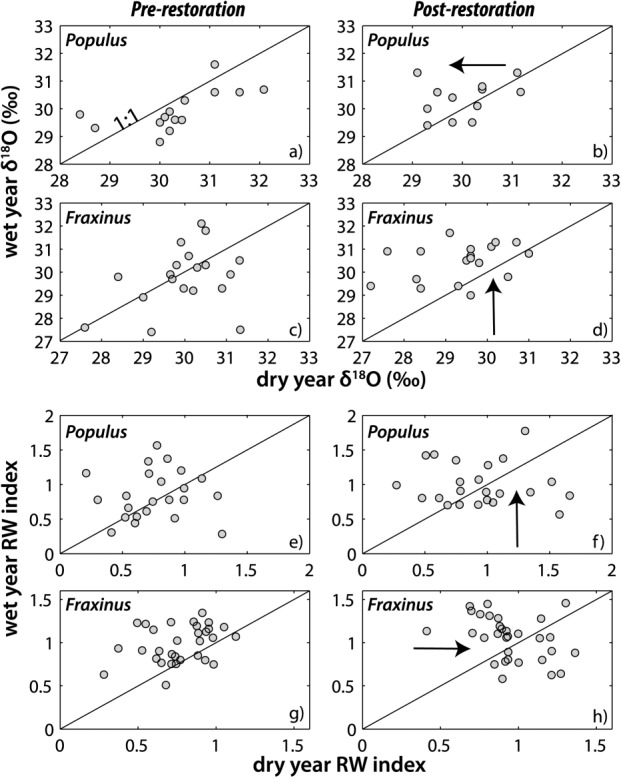
Individual oxygen isotope ratios and dimensionless growth for each species in wet versus dry years before and after the flow restoration. (a) Pre-restoration *Populus δ*^18^O; (b) post-restoration *Populus δ*^18^O; (c) pre-restoration *Fraxinus δ*^18^O; (d) post-restoration *Fraxinus δ*^18^O; (e) pre-restoration *Populus* growth; (f) post-restoration *Populus* growth; (g) pre-restoration *Fraxinus* growth; and (h) post-restoration *Fraxinus* growth. Supporting statistics are provided in Tables 1 and 2. Arrows indicate the approximate direction of change.

The results from all *δ*^18^O and growth comparisons between groups of trees (cohorts) are presented in Figures 7–12 and significant (*p* < 0.05) differences between cohort distributions (via K-S) are listed in Tables 1 and 2, along with K-S statistics and sample sizes for each comparison. Significant differences in cohort means for individual years (greater than two standard errors around the mean) are indicated by stars in Figures 10. Significantly higher median values for particular cohorts (via Wilcoxon rank sum test) are indicated by shading in the two tables.

**Table 1:** Significantly (*p* < 0.05) Different Variables (Isotopes and Growth)^a^

Variable 1 (V1)	Variable 2 (V2)	K-S stat	*p* Value	n1/n2	V1 Source *δ*^18^O (‰)^b^	V2 Source *δ*^18^O (‰)^b^	V1 − V2 (‰)^b^
*Joint δ*^18^*O*							
Mean *Populus*	Mean *Fraxinus*	0.43	2.9E-02	26/33			
s.d. *Populus*	s.d. *Fraxinus*	0.48	1.1E-02	26/33			
s.d. high *Populus*	s.d. high *Fraxinus*	0.52	3.6E-03	11/18			
s.d. high gravel *Populus*	s.d. high gravel *Fraxinus*	0.43	2.9E-02	9/15			
High gravel *Populus* snow	High gravel *Fraxinus* snow	0.70	1.7E-02	7/10	−9.43	−7.70	−1.73
*Populus* 2001–2010	*Fraxinus* 2001–2010	0.18	3.3E-03	163/109	−7.58	−8.61	1.03
High *Populus* 2001–2010	High *Fraxinus*2001–2010	0.31	5.1E-04	66/106	−7.58	−8.48	0.90
High gravel *Populus* 2001–2010	High gravel *Fraxinus* 2001–2010	0.26	3.9E-03	73/108	−7.58	−8.34	0.76
Shallow *Populus* 2001–2010	Shallow *Fraxinus* 2001–2010	0.35	9.0E-03	73/30	−7.85	−9.17	1.32
Shallow *Populus* 1990–1999	Shallow *Fraxinus* 1990–1999	0.41	2.0E-03	68/27	−7.87	−8.90	1.03
*Joint Growth*							
s.d. *Populus*	s.d. *Fraxinus*	0.86	8.9E-08	25/31			
s.d. high *Populus*	s.d. high *Fraxinus*	0.57	1.1E-03	11/18			
s.d. low *Populus*	s.d. low *Fraxinus*	0.62	2.9E-04	11/9			
s.d. shallow *Populus*	s.d. shallow *Fraxinus*	0.81	5.5E-07	9/3			
s.d. deep *Populus*	s.d. deep *Fraxinus*	0.67	7.1E-05	8/10			
s.d. gravel high *Populus*	s.d. gravel high *Fraxinus*	0.52	3.6E-03	9/14			
s.d. gravel low *Populus*	s.d. gravel low *Fraxinus*	0.52	3.6E-03	8/6			
Gravel high *Populus* post-wet	Gravel high *Fraxinus* post-wet	0.60	2.3E-02	9/14			
Gravel low *Populus* pre-dry	Gravel low *Fraxinus* pre-dry	0.75	1.9E-02	8/6			
Gravel low *Populus* rain	Gravel low *Fraxinus* rain	0.71	3.2E-02	8/6			
High *Populus* post-wet	High *Fraxinus* post-wet	0.53	2.9E-02	11/18			
Deep *Populus* post-dry	Deep *Fraxinus* post-dry	0.63	3.4E-02	8/10			
*Populus* 2001–2010	*Fraxinus* 2001–2010	0.12	3.8E-02	239/296			
Deep *Populus* 2001–2010	Deep *Fraxinus* 2001–2010	0.22	2.1E-02	78/99			
*Populus δ*^18^*O*							
*Populus* pre-dry	*Populus* pre-wet	0.44	3.6E-02	25/16	−7.75	−7.98	0.23
Low *Populus* pre-dry	Low *Populus* pre-wet	0.65	4.0E-02	11/11	−8.15	−8.53	0.38
Gravel high *Populus* pre-dry	Gravel high *Populus* pre-wet	0.71	1.7E-02	9/7	−7.47	−7.71	0.24
Deep *Populus* snow	Shallow *Populus* snow	0.86	3.8E-02	3/7	−7.91	−9.16	1.25
Gravel high *Populus* snow	Gravel low *Populus* snow	0.86	1.9E-02	7/4	−9.43	−7.91	−1.52
Gravel high *Populus* snow	Gravel high *Populus* rain	0.69	4.8E-02	7/6	−9.43	−7.10	−2.33
Low *Populus* 2001–2010	High *Populus* 2001–2010	0.38	7.5E-05	67/66	−8.55	−7.58	−0.97
Low gravel *Populus* 2001–2010	High gravel *Populus* 2001–2010	0.43	5.0E-05	73/45	−8.69	−7.58	−1.11
*Populus Growth*							
Low *Populus* snow	Low *Populus* rain	0.80	1.2E-03	10/10			
Deep *Populus* pre-dry	Shallow *Populus* pre-dry	0.68	1.8E-02	8/10			
*Fraxinus δ*^18^O							
Deep *Fraxinus* snow	Shallow *Fraxinus* snow	1.00	3.3E-02	3/3	−7.70	−8.80	1.10
Low *Fraxinus* 1990–1999	High *Fraxinus* 1990–1999	0.24	2.5E-02	58/101	−8.49	−7.93	−0.56
Deep *Fraxinus* 1990–1999	Shallow *Fraxinus* 1990–1999	0.48	4.5E-04	45/27	−7.93	−8.90	0.97
*Fraxinus Growth*							
*Fraxinus* pre-dry	*Fraxinus* pre-wet	0.45	2.2E-03	31/31			
High *Fraxinus* pre-dry	High *Fraxinus* pre-wet	0.56	4.3E-03	18/18			
*Fraxinus* post-dry	*Fraxinus* post-wet	0.42	5.7E-03	31/31			
Low *Fraxinus* post-dry	Low *Fraxinus* post-wet	0.67	1.9E-02	9/9			
Shallow Fraxinus post-dry	Shallow Fraxinus post-wet	1.00	3.3E-02	3/3			
*Fraxinus* snow	*Fraxinus* rain	0.48	8.1E-04	31/31			
High *Fraxinus* snow	High *Fraxinus* rain	0.44	3.9E-02	18/18			

a Shaded cells indicate variables with significantly higher medians (measured by Wilcoxon rank sum test (*p*<0.05)). No shading means the two variables are drawn from statistically distinct distributions, but neither variable has higher median.

b Source water *δ*^18^O to root zone back-calculated using model of *Barbour et al*. [2004]. See supporting information.

**Table 2:** Significantly (*p* < 0.05) Different Variables (Isotopes and Growth) Pre-restoration Versus Post-restoration

Variable 1 (V1)	Variable 2 (V2)	K-S stat	*p* Value	n1/n2	V1 Source *δ*^18^O (‰)^a^	V2 Source *δ*^18^O (‰)^a^	V1 − V2 (‰)^a^
*Populus δ*^18^*O*							
Low gravel *Populus* pre-dry	Low gravel *Populus* post-dry	0.88	0.03	8/3	−8.02	−9.59	1.57
*Populus Growth*							
Low *Populus* pre-dry	Low *Populus* post-dry	0.60	0.03	10/10			
Shallow *Populus* pre-dry	Shallow *Populus* post-dry	0.60	0.03	10/10			
Low gravel *Populus* pre-dry	Low gravel *Populus* post-dry	0.63	0.05	8/8			
*Fraxinus δ*^18^*O*							
*Fraxinus* pre-dry	*Fraxinus* post-dry	0.39	0.02	32/26	−8.36	−9.53	1.17
High gravel *Fraxinus* pre-dry	High gravel *Fraxinus* post-dry	0.58	0.01	15/14	−8.09	−8.72	0.63
*Fraxinus Growth*							
*Fraxinus* pre-dry	*Fraxinus* post-dry	0.39	0.01	31/31			
High *Fraxinus* pre-dry	High *Fraxinus* post-dry	0.56	0.00	18/18			
Deep *Fraxinus* pre-dry	Deep *Fraxinus* post-dry	0.55	0.05	11/11			
High gravel *Fraxinus* pre-dry	High gravel *Fraxinus* post-dry	0.57	0.01	14/14			

a Source water *δ*^18^O to root zone back-calculated using model of Barbour et al. [2004]. See supporting information. Shaded cells indicate variables with significantly higher medians (measured by Wilcoxon rank sum test (*p* < 0.05)). No shading means the two variables are drawn from statistically distinct distributions, but neither variable has higher median.

### 4.1. Overall Interspecific Comparisons

Comparing all trees across the two species, year-on-year mean *δ*^18^O values for *Populus* and *Fraxinus* were drawn from significantly different distributions (Table 1), but the median values of these cohort means over the 21 year period are statistically similar. That is, there were annual changes in each species that suggest they were accessing distinct water sources in particular years which fluctuate in isotopic composition, but neither is consistently higher or lower than the other. However, the SD in the *δ*^18^O time series is significantly higher for *Fraxinus* (SD > 1.1‰) than for *Populus* (Table 1). The higher *δ*^18^O variability in *Fraxinus* for all trees studied, especially those with high floodplain and gravel elevations, corresponds to lower variability in growth for *Fraxinus* than for *Populus* over all cohorts for this decadal period (Table 1 and Figures 10). This result is consistent with isotopic and growth results for these species from prior work along a Rhône tributary, which hypothesized that *Fraxinus* is more able to switch source water than *Populus* in order to maintain more steady year-to-year growth [*Singer et al.*, 2013].

### 4.2. Intraspecific Comparisons: High Versus Low Floodplain Elevation, Shallow Versus Deep Soils, and High Versus Low Gravel Elevation

There are no overall significant differences in *δ*^18^O or growth for the following cohorts of either species over the 21 year period: high versus low elevation, shallow versus deep soils, and high versus low gravel elevation (Figures 10). There are, however, significant differences between these cohorts for particular years, as well as before and after the flow restoration. These will be discussed in the relevant sections below.

### 4.3. Wet Versus Dry Hydrologic Years

To isolate the role of climate on water availability to the root zone for each species, we selected one wet and one dry year for each of two segments of the time series—before and after the flow restoration. Wet years are characterized by relatively high *Q* and *P* for the year and for the growing season, and vice versa for dry years (Figure 4). The K-S test yielded significant differences between wet versus dry-year *δ*^18^O in *Populus* trees during the pre-restoration period (before 2000), which is selectively expressed at low floodplain surface elevations and at high gravel elevations (Table 1 and Figures 7b, 9b, and 9d), though none of these comparisons yielded statistically higher/lower median values. That is, we did not find differences in the medians of samples assembled as compilations of all available trees for each cohort for wet versus dry years. However, mean values of *δ*^18^O for these *Populus* cohorts were enriched in 1998 compared with 1999 (visually, if not statistically, Figures 7 and 9). In order to estimate the magnitude of isotopic differences in source water (rather than to accurately estimate the absolute isotopic value of the water used by the trees), we back-calculated *δ*^18^O in the source water used by *Populus* trees (supporting information) using these mean cohort isotopic values in cellulose within the Barbour model, along with average observed climatic parameters, and field-measured conductance/transpiration rates for wet versus dry years [*Lambs et al.*, 2006]. We found the largest (yet modest) calculated difference in source waters between 1998 and 1999 for low *Populus*, which indicated slightly more depleted *δ*^18^O in the pre-restoration wet year compared with the dry year (by −0.4‰). The direction of these computed differences in source water is the same for the other *Populus* cohorts (Table 1; supporting information), which indicates minor differences in interannual source water for these cohorts for the pre-restoration period.

The isotopic differences for *Populus* are also not significant between the wet and dry years selected for the period after the flow restoration (2001 and 2005, Figure 4). Additionally, none of these wet versus dry year differences in water source had a significant effect on growth for *Populus* (Table 1), although the prerestoration wet year (1999) clearly generated substantially higher growth for low *Populus* than the dry (1998) year (Figures 8b, 10b, and 10d). Growth in the dry year for the deep cohort was significantly different from that in the shallowly rooted cohort even though the medians for these cohorts were statistically similar (Table 1).

*Fraxinus* cellulose exhibited no significant differences in *δ*^18^O between wet and dry years before or after the flow restoration. However, in terms of growth, there are many significant differences for this species. *Fraxinus* grew faster in the wet years (both 1999 and 2001) than the dry ones, and particularly at high floodplain elevations for 1999. The low and shallowly rooted cohorts of trees for this species had significantly different distributions of growth between 2001 and 2005, but their medians were statistically similar (Table 1). Although there were no significant differences in *δ*^18^O for particular wet or dry years between species, *Fraxinus* grew significantly faster than *Populus* in: 2001 (wet) for high and high gravel cohorts; 2005 (dry) for deeply rooted trees; and in 1998 (dry) for low gravel cohorts (Table 1 and Figures 8 and 10).

### 4.4. Rainfall Versus Snowmelt

Hydrologic years characterized by strong spring rains versus high spring snowmelt potentially provide opportunities to isolate the isotopic signature of two distinct water sources within tree rings and for corresponding tree growth. For *Populus*, we find differences in *δ*^18^O between the spring snowmelt (1995) versus rainfall (1997) years in trees rooted at high gravel elevations (Table 1), although the medians for these cohorts are statistically indistinguishable, apparently due to the high SD for high gravel *Populus* in 1997 (Figure 9d). However, it is evident that *Populus* trees rooted at high gravel elevations used isotopically depleted water in the snowmelt year and relatively enriched water in the rainfall year. Our computations with the Barbour model indicate that source waters used by high gravel *Populus* were depleted by −2.3‰ in 1995 compared to 1997, which is consistent with a colder source of precipitation contributing to available water at the root zone or at least partial use of shallow phreatic groundwater. Furthermore, during this relatively high snowmelt year (Figure 4), back-calculated water sources incorporated into high gravel *Populus* cellulose were significantly depleted compared to those at low gravel elevations (by −1.5‰). The shallowly rooted trees also apparently used more depleted water compared with deeply rooted ones (by −1.3‰, Table 1; supporting information), which accessed a water source with the same isotopic signature as those at low gravel elevations. In terms of growth, low *Populus* trees had significantly higher growth during 1995 than in 1997 (Table 1 and Figure 8b).

*Fraxinus* tree ring *δ*^18^O for deep versus shallow rooting trees was significantly different for the snowmelt year. Although the medians are not significantly different, it is apparent that shallow soils provided a more depleted source of water (by −1.1‰) to *Fraxinus* (consistent with the direction of difference and the back-calculated value we found for *Populus*) in this year of low spring rainfall (Table 1 and Figures 4 and 9a). Growth was significantly higher in the snowmelt year versus the rainfall year for all *Fraxinus* trees, especially those at high floodplain elevations.

Over all cohorts, the high rainfall year provided more enriched water to *Populus* than the snowmelt year, and the snowmelt year delivered a more enriched water source to *Fraxinus* than the rainfall year, and these differences in water sources may have enabled both species to maintain similar growth for these distinct hydrologic years (Figure 11).

### 4.5. Pre-flow Versus Post-flow Restoration

The flow restoration of 2000 raised the low water table surface by ∼0.5 m (Figure 6), which has the potential to impact the partitioning of floodplain water and which may manifest differently between wet versus dry years. Our comparisons of wet and dry years before and after the flow restoration at Pierre-Bénite are approximate in that it is unlikely that the precise hydrologic conditions within a particular year (i.e., the *P* and *Q* regimes) will be reproduced. However, these approximate comparisons of wet and dry years reveal some general changes in hydrologic partitioning that have likely occurred in response to the flow restoration in 2000 (Figure 12).

For *Populus*, we found significant differences in dry-year *δ*^18^O and growth for trees at low gravel elevation. These differences indicate that the low gravel *Populus* used a more depleted source of water in the post-restoration dry year of 2005 (by −1.6‰, Table 2) and grew significantly faster that year than in 1998 (Table 2 and Figures 9d and 10d). In addition, low elevation and low gravel elevation *Populus* cohorts were significantly depleted in *δ*^18^O compared with their high elevation and high gravel counterparts for the entire post-restoration period (2001–2010), which suggests a new water source used by these cohorts that enabled them to grow faster (Table 2). Perhaps in a related manner, overall *Populus* growth between high and low elevation trees is uncorrelated before the restoration, but becomes more coordinated for particular years (2007–2009) after its implementation. Although there is no significant difference in *δ*^18^O for the low cohort before and after the flow restoration (*p* = 0.07), the isotopic water source appears to be trending downward (Figures 7b and 9d). Given the already low *p* value for the K-S test, the pre-restoration and post-restoration differences will likely become statistically significant for all low *Populus* trees following a few more years of access to elevated summer flows. However, median *δ*^18^O for the low gravel elevation cohort is already statistically more depleted after the restoration than before it (*p* = 0.0124, *T* = 0.003, n1/n2 = 48/45), while it is more enriched for the high gravel *Populus* cohort after restoration (*p* = 0.0497, *T* = 0.004, n1/n2 = 69/73). These factors suggest source switching for particular cohorts of *Populus* within the same floodplain site.

For *Fraxinus*, we detected significantly depleted dry-year isotopic values in all trees for 1998 compared with 2005, which is expressed prominently at high gravel elevations. *Fraxinus* growth was also significantly higher for the post-restoration dry year (2005) for several cohorts. There are systematic differences in *δ*^18^O between low versus high and shallow versus deep *Fraxinus* cohorts for the pre-restoration time period, with low and shallow cohorts apparently obtaining more depleted water that the other cohorts (Table 1; supporting information). There are no significant differences between *Fraxinus* cohorts after the restoration, but there is an increase in the post-2000 SD in *δ*^18^O (Figure 9a). However, *Fraxinus* adjusted toward less coordinated growth between high versus low cohorts after 2000 (correlation adjusts from significant value of *ρ* = 0.92; *p* = 0.00015 before restoration to *ρ* = 0.21; *p* = 0.56 after restoration; Figure 8a), which also occurred for high gravel versus low gravel cohorts.

Both *Populus* and *Fraxinus* exhibit apparent shifts in their water sources and growth between the wet and dry years after the restoration. This occurs largely toward more isotopically depleted water for *Populus* in the dry year and toward more enriched water in the wet year for *Fraxinus* (Table 2 and Figures 7, 9, and 12a–12d). Perhaps, in a related manner, growth has modestly increased in the wet year for *Populus* and in the dry year for *Fraxinus*. However, this result is equivocal because different cohorts of a particular species may offset each other (e.g., dry-year growth for shallow rooting *Populus* has increased, while it has declined for deeply rooted ones). However, it has had an important impact to dry-year growth globally for *Fraxinus*, which increased substantially for many cohorts (Table 2 and Figures 8, 10, and 12e–12h). Again, we can only draw limited conclusions from these comparisons because the pre-restoration versus post-restoration wet or dry hydrologic years would not be expected to have identical floodplain water partitioning. However, one notable result is that many cohorts of *Populus* retain significantly more enriched water signatures in their cellulose for the post-restoration period than their *Fraxinus* counterparts (Table 1). These cohorts are those which would not be expected to have access to the new phreatic water source contributed by the flow restoration (high, high gravel, shallow), and have apparently adapted to a more enriched source of floodplain water.

## 5. Discussion

This study investigated ecohydrology within a forested riparian corridor designed to assess the influence of various physical controls on water partitioning between different floodplain water reservoirs over a range of hydrologic years. Our detailed approach used trees as annual integrators of the hydrologic cycle, particularly in terms of water availability at the root zone, and its uptake and incorporation into tree ring cellulose. We compared normalized annual tree ring widths and *δ*^18^O against various local parameters (soil depth, floodplain surface elevation, gravel elevation, and local water *δ*^18^O) that differ for groups of trees. We further assessed these physical controls in the context of characteristic hydrologic years over several decades, which might be expected to produce strong differences in water partitioning, especially before and after major human modification of the local water balance. A few interesting observations have emerged from this analysis that warrant further discussion.

First, the observed differences in *δ*^18^O within tree ring cellulose between various cohorts for each species can be considered in terms of the relationship between contributing water sources and the local conditions that affect the particular isotopic mix of source water and its availability at the root zone (Figure 1). In general, a lower floodplain is inundated more frequently by either a rising water table or by overbank flooding from the river, even if precipitation is the same as for a higher plain. However, the surface elevation is not directly relevant to tree roots because they penetrate to different depths depending on tree physiology and textural conditions of the substrate. *Singer et al*. [2013] found that *Fraxinus* roots do not penetrate through gravel substrates, while *Populus* roots do, and *Fraxinus* has mostly shallower roots compared to *Populus* within the same plots [*Sánchez-Pérez et al*., 2008]. Therefore, the relationship between soil depth and gravel elevation may be a more direct control on tree root access to floodplain water reservoirs. For high elevation trees of both species, mean gravel elevation is typically ∼2 m below the floodplain surface, while it is ∼1 m below for low trees (Figure 5a). Gravel elevation in the substrate below high trees is rarely exceeded by river stage (i.e., every several years, Figure 5b). However, water table rise and fall associated with hyporheic flow contributions and/or regional aquifer flow is typically slower and less peaked than river stage, such that shallow groundwater tables rarely exceed even the gravel elevation of low trees, much less that of the high ones (Figure 6). This means that *Fraxinus* trees have virtually no access to phreatic water table, apart from the lowest floodplain trees [*Dufour and Piegay*, 2008], while *Populus* have extremely variable access to shallow phreatic water across the study site. This is in part due to different rooting strategies.

These physical factors must be considered as constants at each tree location when addressing the impacts of annual fluctuations on partitioning of water between floodplain storage reservoirs and corresponding water availability to trees. However, the partitioning of water and its isotopic signature may also vary dramatically year on year due to different combinations of inputs to these storage reservoirs and nonstationary isotopic signatures for each [*Alstad et al.*, 2008; *Brooks et al.*, 2010]. It is likely that these local physical controls on access to annually varying water sources produces, above some threshold of water availability, poor correlation between isotopic signatures of water and climatic variables for cohorts of floodplain trees [*Leffler and Evans*, 1999]. In other words, without controlling for these local variables (in addition to the relevant climatic and water uptake variables), the signal-to-noise ratio in isotopic response to water availability is reduced, especially in the absence of detailed information on water table elevation at the location of each tree.

Second, comparing isotopes in cellulose for the two species overall, *δ*^18^O in *Fraxinus* had higher variability over the time series, whereas *Populus* was more enriched in the post-restoration period than *Fraxinus*, particularly at high floodplain surface elevations, shallow soils, and at high gravel elevations (Table 1). Our calculations (supporting information) suggest the latter enrichment in source waters used by *Populus* could be as much as +1.3‰ for trees rooted in shallow (<1 m) soils. This is striking considering these two species are rooted in the same soils and both presumably have limited access to phreatic water at these rooting locations. So what is the rationale behind this enrichment? When precipitation infiltrates into soils, evaporation near the soil surface typically makes the remaining water heavier isotopically, but this effect diminishes with infiltration depth, such that vertical gradients in *δ*^18^O may be observed [*Gazis and Feng*, 2004; *Hsieh et al.*, 1998; *Lambs and Berthelot*, 2002]. Furthermore, seasonal changes in the source precipitation may affect the initial value of *δ*^18^O delivered to the vadose zone and infiltrating water may interact with antecedent moisture, such that the concentration of *δ*^18^O at the root zone of a particular species develops as a volumetrically weighted average of contributing sources. Moreover, in addition to vertical translatory flow where new water replaces older water in soil pores [*Horton and Hawkins*, 1965], recent work demonstrated that water infiltrating from winter rains may fill up small pore spaces first, where it is tightly bound and retained until the spring growing season, at which point this water may become available to tree roots of Mediterranean trees (even after larger pores drain) [*Brooks et al.*, 2010]. Based on this background understanding, there are several plausible explanations for the relative *Populus* cellulose enrichment in the period 2001–2010

1. Since these cohorts are rooted at locations where they may become easily stranded from phreatic water, they might instead rely on an isotopically enriched source of water near the soil surface that develops due to warmer spring precipitation and to evaporative enrichment. However, since *Fraxinus* also has access to vadose zone water, it may be that the two species are using different components of the available soil water. For example, *Populus* cellulose enrichment could emerge because of investment in a higher density of near-surface roots than *Fraxinus*, allowing it access to relatively enriched water due to spring/summer evaporation (Figure 1). Since *Populus* typically invest in deep root growth, they have much smaller roots in the vadose zone, compared with co-occurring *Fraxinus* trees (see *Singer et al*. [2013, Figure 6], which shows rooting differences between these species). However, these small near-surface roots in *Populus* are relied upon to obtain water particularly in the upper 30 cm of the soil [*Lambs and Berthelot*, 2002], whereas *Fraxinus* likely obtains water from depths throughout the vadose zone, potentially yielding a more depleted oxygen isotopic signature.

2. If the infiltrating water does in fact get stored in small pores, disconnected from translatory flow yet available for subsequent extraction by vadose zone roots, this should occur at various elevations in the soil column. In other words, early autumn/winter rains that have a relatively depleted isotopic signature (Figure 3) would be responsible for filling up small soil pores throughout the draining soil column. However, water stored in such fine pores closer to the soil surface would likely undergo evaporative enrichment in various periods through the hydrologic year, and especially during the warm spring/summer months. As a result, water extracted from these pores would contain more enriched water than that extracted from small pores at deeper soil levels, even though the water may derive from the same rainstorms. *Populus* certainly exhibits higher rates of sap flow [*Lambs and Muller*, 2002; *Sánchez-Pérez et al*., 2008], which may enable this species to obtain tightly bound water through shallow vadose zone roots at low soil matric potentials, especially as a “last-chance” water source, once deeper reservoirs become exhausted [*Snyder and Williams*, 2000]. However, this vadose zone water source may not satisfy the high demand for this species [*Lambs and Muller*, 2002], due to insufficient dimorphic rooting [*Dawson and Pate*, 1996], creating negative consequences for *Populus* growth [*Lambs et al.*, 2006] compared to *Fraxinus* especially in dry years (Table 1 and Figures 8 and 10). These factors suggest an acute vulnerability for *Populus* associated with increased drought conditions that lower regional water tables [*Amlin and Rood*, 2003; *Singer et al.*, 2013]. However, it appears that *Populus* in the Pierre-Bénite site may have adapted to infrequent phreatic water supply by growing more shallow roots to access this relatively enriched water source in order to maintain sufficient interannual growth (Figures 1, 9, and 10 and Table 1). It is not clear whether this could impact water availability to *Fraxinus*. In any event, these two species for particular cohorts have developed distinct sources waters.

3. If transpiration/conductance for *Populus* declined dramatically in this latter period, leaf water could have progressively enriched the preserved tree ring isotopic signature in these trees (based on manipulations of the Barbour model). However, there is no evidence for this, since annual growth rates have not changed dramatically over this period for the species and there have been no significant climatic differences to drive such changes in tree physiological response.

Third, the differences in isotopic signatures between the snowmelt (1995) and rainfall (1997) years suggest that *Populus* trees generally used more isotopically enriched water in 1997, whereas *Fraxinus* used more enriched water in 1995 (Figure 11). However, closer examination of individual cohorts yields more insight into the floodplain ecohydrology during these contrasting water years. During the high snowmelt year, *Fraxinus* trees rooted in shallow soils, high surface elevations, and low gravel elevations evidently used a more depleted water source than their counterpart trees in deep soils, low elevations, and high gravel elevations (Figures 7a, 9a, and 9c), whereas *Populus* trees rooted at high gravel elevations accessed more depleted water than those at low gravel elevations during this year (Figure 9d). The former result is intuitive; most *Fraxinus* cohorts cannot typically access the hyporheic waters associated with spring snowmelt (Figure 9c) because their roots do not penetrate gravel, so significant differences in cohort *Fraxinus* isotopic signatures for 1995 are likely a function of soil depth. In this case of low growing season rainfall (Figure 4), trees rooted in deep soils have access to more enriched water from the vadose zone (Table 1 and Figure 9a). In 1995, GNIP records for Thonon Les Bains (no records exist for Avignon for this year) show that there was anomalously high rainfall in May (1.9 cm) containing an unusually depleted isotopic signature for this time of year (−10.6‰). The remainder of the growing season had very low rainfall with more enrichment in *δ*^18^O. Thus, it is possible that *Fraxinus* trees rooted in shallow soils and at high gravel elevations used this early growing season rainfall and thus recorded a more depleted isotopic water source compared with cohorts obtaining a greater mix of growing season precipitation (through a deeper soil profile). Since *Populus* trees in shallow soils and those at high gravel positions had a similar value of back-calculated source water to that of shallow-rooting *Fraxinus* (within 0.4‰), they apparently used the same source of water, which was 1.7‰ more depleted (likely phreatic source) compared with its *Fraxinus* equivalent (Table 1; supporting information). In contrast, the deeply rooted and low gravel position cohorts of both species accessed a more enriched water source, indicative of the larger mix of annual precipitation. It is not clear whether there was enough hyporheic flow in this snowmelt year to raise the water table to elevations reachable by any trees in Pierre-Bénite, but we speculate that high gravel *Populus* gained access to this phreatic water source (Figure 6; supporting information).

In the year 1997, *Q* was reduced and spring rains dominated growing season floodplain hydrology. While *Fraxinus* did not exhibit any significant differences between the various cohorts, *Populus* at high floodplain elevations, high gravel elevations, and those rooted in deep soils apparently took advantage of enriched soil moisture from relatively warm and high growing season rains (Figures 3 and 9d). This source of water may have allowed these cohorts to maintain their growth in high spring rainfall years and more steady annual growth overall, compared to their low gravel elevation cohort (Figure 10d and Table 1). Again, it appears that the water table was not available to any of our trees in 1997 (Figure 6).

Fourth, the restoration has certainly had an important impact on some riparian trees at this site, especially *Populus*. The effect of the restoration is evident for high versus low trees of this species (Figure 7b), but even more so for high versus low gravel elevations (Figure 9d). These isotopic signals diverge as trees at low floodplain and gravel elevations accessed increasingly depleted water from the shallow phreatic zone (Table 1), allowing much more elevated and consistent growth for this species in dry years (Figure 10d). Annual differences in mean growth and *δ*^18^O between *Populus* cohorts at high versus low elevations and high versus low gravel elevations support our conclusions about the effect of the flow restoration. Increases in yearly differences in *δ*^18^O for high versus low *Populus* after the restoration yielded fewer differences in annual growth (Figures 7b and 8b). Overall, the flow restoration appears to have had an impact on source water availability to riparian trees at Pierre-Bénite, especially *Populus*.

The availability of water to riparian trees is often discussed in terms of end-member sources of phreatic water [*Busch et al.*, 1992; *Dawson and Ehleringer*, 1991] versus vadose zone water [*Plamboeck et al.*, 1999]. However, in riparian environments in particular, modest variations in driving hydrology based on climatic fluctuations may notably influence the partitioning of water between these sources, especially in floodplains with heterogeneous structure (i.e., in terms of sedimentary architecture and elevation). Thus, the isotopic signature of water preserved in vegetation is likely to fluctuate annually based on water availability alone [*Walker and Richardson*, 1991] and will vary across individual stands of forest. These variations in tree ring *δ*^18^O are ultimately reflective of complex interactions between driving hydrology, which varies by annually and seasonally by magnitude, timing, and phase of precipitation, and the local conditions of topography and soil texture that influence the movement of water between the various saturated and unsaturated floodplain storage reservoirs (Figure 1). Such annual differences may be expected to undergo substantial and unpredictable changes, as climatic shifts are expressed in particular regions. For example, the warming and drying of basins such as the Rhône will affect precipitation regimes, evaporation rates, and thus antecedent moisture in floodplain forests [*Singer et al.*, 2013].

Anthropogenic influence on river-floodplain corridors may also dramatically impact the partitioning of water between these storage reservoirs. The influence of hydrologic modifications on streamflow regimes are well documented throughout the world [*Nilsson et al.*, 2005] and their influence may propagate or dissipate through the fluvial network [*Singer*, 2007]. In addition, impacts to flow regimes due to water extraction [e.g., *Maheshwari et al.*, 1995] or even large-scale rehabilitation scenarios aimed at improving river-floodplain interaction and functioning [e.g., *Singer and Dunne*, 2004,^2006^] are likely to change streamflow regimes, hyporheic flow, and regional shallow groundwater tables. As these changes influence the annual availability of water in floodplain storage reservoirs, we should expect impacts to riparian forests [*Amlin and Rood*, 2003; *Rains et al.*, 2004; *Snyder and Williams*, 2007]. However, the prediction of such ecohydrologic responses requires detailed study into the imprint of human alteration of hydrology on top of inherent (or even shifting) climatic variability and local topographic and sedimentary controls that influence partitioning of water in floodplains.

## 6. Conclusions

The two-species comparison for analyzing floodplain ecohydrology yields new insight into topographic and hydrologic controls on tree water availability and growth, especially when considering separately the climatic versus anthropogenic versus local physical controls on water availability to the root zone after constraining the potential relative roles of leaf-water enrichment versus source waters. There is still much to be learned about the integration of isotopic signatures into tree rings that reflect annually and seasonally varying water sources. Several factors were presented that challenge interpretations of past climate or even of contemporary plant-water relations, especially in water-rich floodplains, when viewed at the spatial scale of at least a forest stand and over a period of decades. Further work could explore the oxygen isotopic signatures of water availability and uptake at these scales to better inform predictive models of forest health with respect to climatic, anthropogenic, and physical perturbations.

## Supporting Information

• Read Me

• Dataset 1

• Supporting Figures 1 and 1
