# Supplementary figures and images for: Floodplain ecohydrology: Climatic, anthropogenic, and local physical controls on partitioning of water sources to riparian trees
Source: Water Resour Res. 2014 May 29;50(5):4490–513. doi: 10.1002/2014WR015581 (PMC4258096; doi:10.1002/2014WR015581)

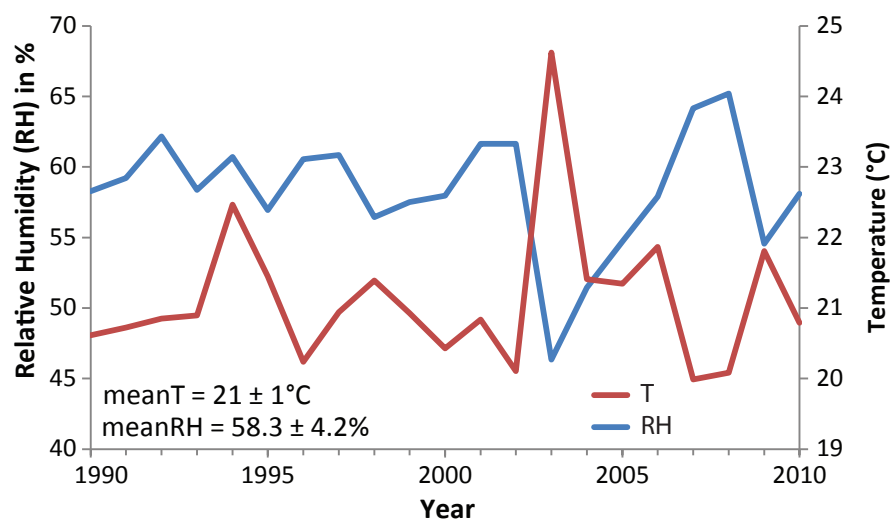

Supplement: Supplementary file 3 — • Supporting Figures 1 and 1 [file wrcr0050-4490-SD3.pdf]

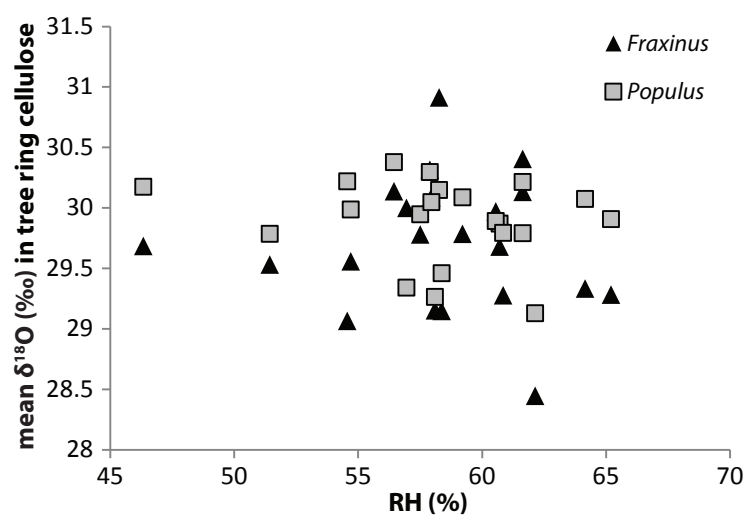

Supplement: Supplementary file 4 — • Supporting Figures 1 and 1 [file wrcr0050-4490-SD4.pdf]
